# Utility of Estimating Glycated Hemoglobin in Sodium Fluoride Tube and by Turbidimetric Immunoinhibition Method: Our Experience in a Tertiary Care Cancer Centre

**DOI:** 10.7759/cureus.81164

**Published:** 2025-03-25

**Authors:** Shrikant Raut, Kalpita Naik, Vinayak Parab, Nikhil Choudhary, Kinjalka Ghosh

**Affiliations:** 1 Department of Biochemistry, Tata Memorial Hospital, Tata Memorial Centre, Mumbai, IND; 2 Biochemistry, Homi Bhabha National Institute (HBNI), Mumbai, IND

**Keywords:** edta tube, glycated hemoglobin (hba1c), high performance liquid chromatography (hplc), sodium fluoride tubes, turbidimetric immunoinhibition

## Abstract

Background

Diabetes mellitus in cancer patients is monitored by measuring blood glucose levels and glycated hemoglobin (HbA1c) collected in sodium fluoride and EDTA tube respectively. This study intends to estimate HbA1c from a sodium fluoride tube and compare it with a standard EDTA tube eliminating the requirement of collecting additional samples in an already challenging venipuncture in cancer patients and also compare HbA1c levels estimated by turbidimetric immunoinhibition with reference high-performance liquid chromatography (HPLC) method.

Methods

This cross-sectional study was conducted at the Department of Biochemistry of Tata Memorial Hospital, Mumbai, India, over a period of five months. Blood samples of patients received in sodium fluoride and EDTA tubes at the same time for estimation of blood glucose and HbA1c respectively were included in the study. HbA1c results by HPLC method on Bio-Rad D10 analyzer were compared with turbidimetric immunoinhibition method on Beckman Coulter AU 5800 analyzer. Data was analyzed using statistical software SPSS version 29.

Results

Blood samples of 384 patients, of which 193 (50.3%) were female and 191 (49.7%) were male with an average age of 55.2 ± 12.4 years were included in the study. The majority of these samples were from 78 (20.3%), 77 (20.1%) and 60 (15.6%) patients with urological cancers, breast cancer and gastrointestinal cancers, respectively. There was no significant difference in the average HbA1c value estimated from EDTA and sodium fluoride tube by HPLC (6.71±1.7 and 6.71±1.7, respectively) and turbidimetric immunoinhibition (6.74±1.6 and 6.72±1.7, respectively) method. A strong and significant correlation was observed in HbA1c values estimated from either tube by both methods (p-value <0.0001).

Conclusion

Our study confirms that HbA1c can be conveniently estimated from a sodium fluoride tube, thereby eliminating the necessity of an EDTA tube for the same and it also observes that HbA1c results by HPLC and turbidimetric immunoinhibition method are comparable and thus can be analyzed by either of the methods interchangeably.

## Introduction

Diabetes mellitus, a metabolic disorder is not only a common disease among cancer patients but also a risk factor for certain solid malignancies [1,2]. Literature suggests that cancer patients with diabetes have higher cancer-related mortality and are at an increased risk of various infections and infection-related morbidity and mortality [2-5]. Thus, making it imperative to treat and monitor diabetes regularly in cancer patients.

The American Diabetes Association and World Health Organization recommend treating and monitoring diabetes by estimating fasting blood glucose (FBG), Post-prandial blood glucose (PPBG), and glycated hemoglobin (HbA1c) [6]. FBG and PPBG samples are routinely collected in tubes containing sodium fluoride (NaF), whereas the HbA1c sample is collected in an EDTA tube. HbA1c is estimated by methods such as high-performance liquid chromatography (HPLC), turbidimetric immunoinhibition (TI), and capillary electrophoresis with a good concordance between the results [7]. Among these methods, HPLC remains the gold standard or reference method as suggested by the National Glycohemoglobin Standardization Program (NGSP) due to the long-term stability and accuracy in HbA1c measurement.

Venipuncture and blood collection can be challenging in cancer patients as they tend to have difficult venous access due to cachexia, advanced age, scarring from repeated intravenous access, and/or repeated administration of chemotherapeutic medications [8]. What if both FBG and HbA1c can be done from the NaF tube? It can not only save the amount of blood collected in critical patients but also save on an additional tube. The purpose of collection in an EDTA tube to prepare hemolysate from red blood cells can also be carried out by NaF thus eliminating the necessity of drawing an additional blood sample from the patient who has already given a sample for estimation of FBG.

There were only a few studies conducted to rule out any interference between the types of blood collection tubes used for HbA1c estimation and the method of estimation [9,10]. Thus, this study aims to evaluate the impact on HbA1c levels estimated in blood samples collected in NaF tubes Vs the standard EDTA tube and also compare HbA1c values measured by TI and HPLC methods in the same patient.

## Materials and methods

The study was conducted at the Department of Biochemistry of Tata Memorial Hospital, Mumbai, India, over a period of five months (November 2023 to March 2024) after obtaining ethical clearance from the institutional ethics committee (Approval number: 4229). A sample size of 384 was calculated by using OpenEpi version 3, an online sample size calculator for the power of >95 % and p-value <0.05.

Inclusion criteria

Blood samples of cancer patients received in the Department of Biochemistry for estimation of both FBG (NaF tube) and HbA1c (EDTA tube) levels at the same time were included in the study.

Exclusion criteria

Insufficient sample, FBG and HbA1c samples of the same patient received at different time intervals and patients with hemoglobinopathies were excluded.

Basic clinical and general information (such as; age, gender, primary disease) of the patient whose blood sample has been included in the study was noted. HbA1c test routinely processed from EDTA tube on Bio-rad Laboratories D10 analyzer by HPLC method was also processed on Beckman Coulter AU 5800 analyzer by TI method for comparison. Samples received in the NaF tube of the same patient for estimation of FBG were also processed for the HbA1c test on both Bio-rad D10 and Beckman Coulter AU 5800 analyzer to rule out any interference between the types of tubes used for HbA1c estimation and method of estimation. All samples were processed only after a successful internal quality control check by using two levels of control.

SPSS version 29 (IBM Corp, Armonk, USA) was used for statistical analysis. Patient demographic and clinical characteristics were summarized using descriptive statistics like mean and standard deviation for continuous variables and frequency and percentage for categorical variables. The strength of association between the HbA1c measurements was analyzed by Pearson’s correlation. Weighted Deming regression and Bland-Altman plot were used for comparison studies. Student’s t-test assessed the mean difference of HbA1c between the tubes and the method of estimation. Statistical significance was considered at 5%.

## Results

The study included blood samples of 384 patients, of which 193 (50.3%) were female and 191 (49.7%) were male with an average age of 55.2 ± 12.4 years. Out of these 384 patients; 78 (20.3%), 77 (20.1%), 60 (15.6%), 50 (13.0%), 47 (12.2%), 42 (10.9%), 20 (5.2%) and 10 (2.6%) were with urological, breast, gastrointestinal, hematological, head and neck, gynecological, thoracic and bone and soft tissue cancers, respectively.

There was no statistically significant difference in the average of HbA1c values estimated from EDTA and NaF tube by HPLC and TI method as seen in Table 1.

**Table 1:** Difference in the average of HbA1c measured in EDTA & NaF tube by HPLC & TI method using student’s t-test. HPLC: High-performance liquid chromatography; TI: Turbidimetric immunoinhibition; NaF: Sodium fluoride

HbA1c (Mean ± SD)	EDTA Tube	NaF Tube	p-value
HPLC method	6.71 ± 1.7	6.71 ± 1.7	0.979
TI method	6.74 ± 1.6	6.72 ± 1.7	0.897

Table 2 depicts a strong and significant correlation in HbA1c values estimated from EDTA and NaF tubes by HPLC and TI methods.

**Table 2:** Correlation of HbA1c in EDTA & NaF tube by HPLC & TI method HPLC: High-performance liquid chromatography; TI: Turbidimetric immunoinhibition; NaF: Sodium fluoride

		Correlation Coefficient	p-value
EDTA v/s NaF tube	HPLC method	0.9969	<0.0001
	TI method	0.9949	
HPLC v/s TI method	EDTA tube	0.9909	<0.0001
	NaF tube	0.9895	

Table 3 depicts Weighted Deming regression output of comparison of HbA1c by HPLC & TI method. All the intercept and slope estimates are close to zero and one respectively along with a statistically significant p-value indicating that the two methods of estimation are comparable by both the tubes.

**Table 3:** Comparison of HbA1c by HPLC & TI method in EDTA & NaF tube HPLC: High-performance liquid chromatography; TI: Turbidimetric immunoinhibition; NaF: Sodium fluoride

		Coefficient		p-value
		Intercept	Slope	
HPLC v/s TI method	EDTA tube	-0.370	1.051	<0.0001
	NaF tube	-0.340	1.049	

**Figure 1:**
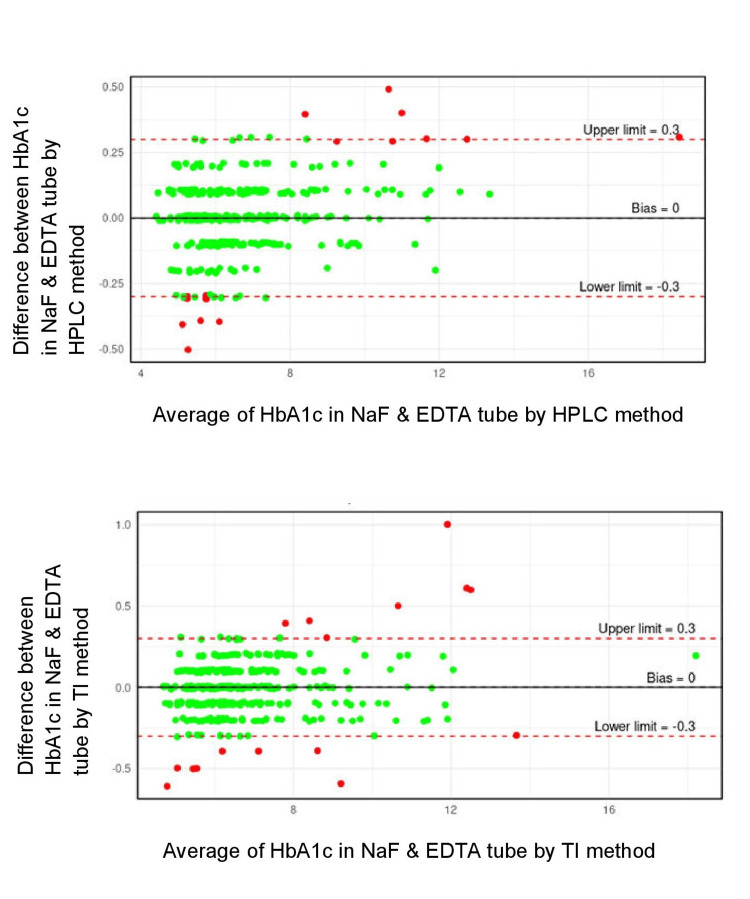
Bland-Altman plot for comparison of HbA1c in NaF & EDTA tube by HPLC & TI method HPLC: High-performance liquid chromatography; TI: Turbidimetric immunoinhibition; NaF: Sodium fluoride

Figure 1 is a Bland-Altman plot for comparison of HbA1c in NaF and EDTA tubes by HPLC and TI method. It shows 96% agreement in HbA1c values between NaF and EDTA tube by HPLC and TI method at ± 0.3 limits of agreement.

Considering 6.5% as the cut-off value of HbA1c estimated from the EDTA tube by HPLC method for diagnosis of diabetes mellitus, estimation by the TI method and from the fluoride tube depicted a sensitivity and specificity of 96.3% and 96.9%, and 95.0% and 98.7%, respectively.

## Discussion

Diabetes mellitus remains to be a challenging comorbid condition in patients with cancer. On one end of the spectrum, the advent of new cancer treatment modalities may unmask underlying diabetes or aggravate pre-existing diabetes [11,12]. Whereas, on the other end concurrent complications associated with diabetes in patients with cancer may influence the choice of cancer therapy.

Diagnosis and monitoring of diabetes in cancer patients is no different from any other disease. HbA1c now remains the primary tool in assessing glycemic control [13]. The test is routinely requested along with blood glucose levels, especially FBG and requires blood to be collected in an EDTA tube. If the HbA1c test can be performed from the NaF tube, the need for additional sample collection will be eliminated. In our study, we observed no significant difference in HbA1c estimated in EDTA and NaF tubes and HbA1c results in the two tubes were comparable. HbA1c values also showed a strong and significant correlation between the two tubes. Similar findings were observed by Gayathri Kini et.al, who not only reported commonality in HbA1c values in EDTA and NaF tubes but also observed a strong correlation in HbA1c values in the above-mentioned tubes [14]. Our study findings were also in concordance with Konar et.al [15] and Kalita et.al [16].

There are various methods to estimate HbA1c such as; HPLC, capillary electrophoresis, and TI. In 1996, HPLC was used as a reference method by NGSP to standardize glycated hemoglobin results compared to those obtained from the Diabetes Control and Complications Trial and since then it has remained a gold standard. In our study, we found that HbA1c values in either tube by HPLC and TI method were well comparable and strongly correlated with each other. A similar comparative study by Genc et.al also noted good agreement in HbA1c values between TI and HPLC methods [7]. Gilani et al. compared HbA1c values with HPLC, capillary electrophoresis, and TI method and found that they correlated well [17].

Venipuncture and blood collection in cancer patients can be cumbersome in view of scarring due to repeated intravenous access and cancer cachexia [8]. Monitoring of diabetes mellitus in cancer patients calls for frequent blood testing. Analyzing HbA1c from NaF tube can surely reduce the volume of blood to be collected and additional samples to be drawn in these patients. It can also save the cost to be borne in order to collect samples in an additional tube. Comparable HbA1c results by HPLC and TI method give the flexibility to run the test on either of the analyzers interchangeably. Another advantage of HbA1c results by the TI method being comparable to the reference HPLC method is that the test can be processed simultaneously with other routine biochemistry tests on the same analyzer and a dedicated analyzer for HbA1c may not be required.

The present study had some limitations, such as the exclusion of patients with hemoglobinopathies due to which the effect of hemoglobin variants on HbA1c results could not be assessed, the small sample size, and the comparison of HbA1c values only across HPLC and TI platforms.

## Conclusions

Our study concludes that HbA1c can be conveniently processed from a NaF tube used for the estimation of blood glucose, thereby eliminating an additional sample to be drawn in an EDTA tube in an already challenging venipuncture in cancer patients. We also observed that HbA1c results by the TI method were comparable with the reference HPLC method giving the ease to analyze the test on any compatible routine biochemistry analyzer.

## References

[1] Ling S, Brown K, Miksza JK (2020). Association of type 2 diabetes with cancer: A meta-analysis with bias analysis for unmeasured confounding in 151 cohorts comprising 32 million people. Diabetes Care.

[2] Bjornsdottir HH, Rawshani A, Rawshani A, Franzén S, Svensson AM, Sattar N, Gudbjörnsdottir S (2020). A national observation study of cancer incidence and mortality risks in type 2 diabetes compared to the background population over time. Sci Rep.

[3] Coughlin SS, Calle EE, Teras LR, Petrelli J, Thun MJ (2004). Diabetes mellitus as a predictor of cancer mortality in a large cohort of US adults. Am J Epidemiol.

[4] Saydah SH, Loria CM, Eberhardt MS, Brancati FL (2003). Abnormal glucose tolerance and the risk of cancer death in the United States. Am J Epidemiol.

[5] Liu X, Ji J, Sundquist K, Sundquist J, Hemminki K (2012). The impact of type 2 diabetes mellitus on cancer-specific survival: A follow-up study in Sweden. Cancer.

[6] Zvarova K, Zvarova Z, Callas PW, Malone-Rising D (2013). New estimates of pre-diabetes and type 2 diabetes prevalence in Mexican Quintana Roo. Int J Diabetes Dev Ctries.

[7] Genc S, Omer B, Aycan-Ustyol E, Ince N, Bal F, Gurdol F (2012). Evaluation of turbidimetric inhibition immunoassay (TINIA) and HPLC methods for glycated haemoglobin determination. J Clin Lab Anal.

[8] Merrill VD, Ward MD, Diaz-McNair J, Pickett EA, Duh SH, Christenson RH (2022). Assessing phlebotomy device preference and specimen quality in an oncology outpatient clinic. J Appl Lab Med.

[9] Mailankot M, Thomas T, Praveena P, Jacob J, Benjamin JR, Vasudevan DM (2012). Various anticoagulants and fluoride do not affect HbA1C level. Indian J Clin Biochem.

[10] Sharma B, Sarmah D, Sonker P (2013). Effect of different anticoagulants on HBA1C estimation and its stability. J Lab Physicians.

[11] Hughes J, Vudattu N, Sznol M, Gettinger S, Kluger H, Lupsa B, Herold KC (2015). Precipitation of autoimmune diabetes with anti-PD-1 immunotherapy. Diabetes Care.

[12] Morviducci L, Rota F, Rizza L (2018). Everolimus is a new anti-cancer molecule: Metabolic side effects as lipid disorders and hyperglycemia. Diabetes Res Clin Pract.

[13] Rohlfing CL, Little RR, Wiedmeyer HM (2000). Use of GHb (HbA1c) in screening for undiagnosed diabetes in the U.S. population. Diabetes Care.

[14] Kini G, Yadav A, Reddy R, Mala M, Golla N, Golla M (2022). Efficacy of sodium fluoride as an anticoagulant in the estimation of glycated haemoglobin in diabetic patients: An alternative to EDTA. J Clin Diagn Res.

[15] Konar S, Karmakar A, Gupta S (2020). Can the level of glucose & glycated hemoglobin be estimated from same container?. Int J Cur Res Rev.

[16] Kalita S, Deori R, Goswami R, Bhattacharyya K (2017). Sodium fluoride vacutainer an alternative to EDTA for estimation of glycated hemoglobin. Int J Sci Res.

[17] Gilani M, Aamir M, Akram A, Haroon ZH, Ijaz A, Khadim MT (2020). Comparison of turbidimetric Inhibition Immunoassay, High-Performance Liquid Chromatography, and capillary electrophoresis methods for glycated hemoglobin determination. Lab Med.

